# MIA: Mutual Information Analyzer, a graphic user interface program that calculates entropy, vertical and horizontal mutual information of molecular sequence sets

**DOI:** 10.1186/s12859-015-0837-0

**Published:** 2015-12-10

**Authors:** Flavio Lichtenstein, Fernando Antoneli, Marcelo R. S. Briones

**Affiliations:** 1 Departamento de Informática em Saúde, Escola Paulista de Medicina, Universidade Federal de Sao Paulo, Rua Botucatu, 862, Ed. José Leal Prado, andar térreo, Vila Clementino, CEP 04023-062 Sao Paulo, SP Brazil; 2 Departamento de Microbiologia, Immunologia and Parasitologia, Escola Paulista de Medicina, Universidade Federal de Sao Paulo, Rua Botucatu, 862, Ed. Ciências Biomédicas, 3 andar, Vila Clementino, CEP 04023-062 Sao Paulo, SP Brazil; 3 Laboratory of Evolutionary Genomics and Biocomplexity, Escola Paulista de Medicina, Universidade Federal de São Paulo, Rua Pedro de Toledo, 669, 4 andar L4E, CEP 04039-032 São Paulo, SP Brazil

**Keywords:** Software, Information theory, Entropy, Mutual information, DNA sequences, Species

## Abstract

**Background:**

Short and long range correlations in biological sequences are central in genomic studies of covariation. These correlations can be studied using mutual information because it measures the amount of information one random variable contains about the other. Here we present MIA (Mutual Information Analyzer) a user friendly graphic interface pipeline that calculates spectra of vertical entropy (VH), vertical mutual information (VMI) and horizontal mutual information (HMI), since currently there is no user friendly integrated platform that in a single package perform all these calculations. MIA also calculates Jensen-Shannon Divergence (JSD) between pair of different species spectra, herein called informational distances. Thus, the resulting distance matrices can be presented by distance histograms and informational dendrograms, giving support to discrimination of closely related species.

**Results:**

In order to test MIA we analyzed sequences from *Drosophila* Adh locus, because the taxonomy and evolutionary patterns of different *Drosophila* species are well established and the gene Adh is extensively studied. The search retrieved 959 sequences of 291 species. From the total, 450 sequences of 17 species were selected. With this dataset MIA performed all tasks in less than three hours: gathering, storing and aligning fasta files; calculating VH, VMI and HMI spectra; and calculating JSD between pair of different species spectra. For each task MIA saved tables and graphics in the local disk, easily accessible for future analysis.

**Conclusions:**

Our tests revealed that the “informational model free” spectra may represent species signatures. Since JSD applied to Horizontal Mutual Information spectra resulted in statistically significant distances between species, we could calculate respective hierarchical clusters, herein called Informational Dendrograms (ID). When compared to phylogenetic trees all Informational Dendrograms presented similar taxonomy and species clusterization.

**Electronic supplementary material:**

The online version of this article (doi:10.1186/s12859-015-0837-0) contains supplementary material, which is available to authorized users.

## Background

Genic and intergenic regions in chromosomes have statistically distinct properties. Most intergenic regions behave randomly in regard to nucleotide mutations, besides some special regions like transcription regulatory sites and transposons. On the other hand, most of the genes are highly conserved, especially in regions like promoters, TATA box, exons, splice junctions etc. But, even these conserved regions, and others such as introns, can present many polymorphic single regions as well as two separated regions that present orchestrated mutations in a way as to try to conserve or improve determined phenotype [1–4]. Hereupon, the main goal of this work is to provide methods and tools in order to discriminate closely related species using informational spectrum distances. Here we searched for polymorphic regions seeking out covariation signals by using DNA sequences from *Drosophila* Adh locus. With these sequences we calculated entropy and mutual informational spectra from different closely related species sequences. Thereafter, via Jensen-Shannon Divergence, we calculated distances between these spectra resulting in distance matrices capable of inferring the possibility of species discrimination.

Furthermore, in microorganisms the species definition is coarse. Attempts to measure sequence diversity by informational entropy and speciation have been proposed in Protista [5, 6]. However, these studies suggest that besides information entropy, mutual information could provide a means to access covariation, a central problem in diversifying molecules and species.

**Fig. 1:**
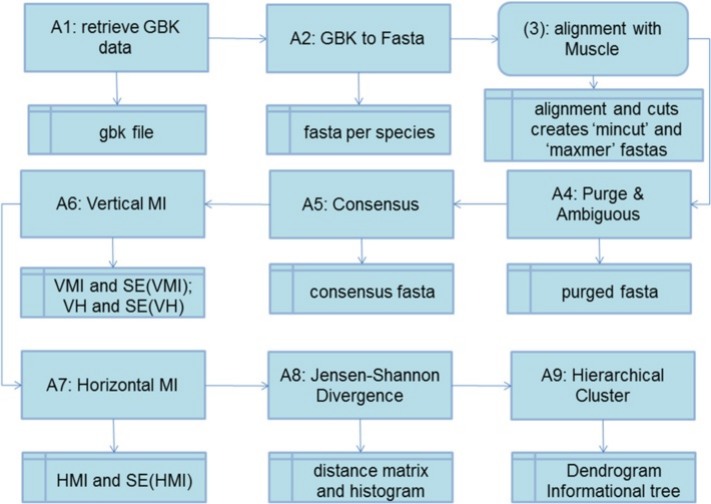
MIA Pipeline: all algorithms (modules) can be seen from A1 to A5 retrieving, preparing and storing sequences; A6 to A7 informational spectra calculation; A8 informational distances calculation and A9 informational dendrograma calculation

Thus, herein we present a computational pipeline called MIA (Mutual Information Analyzer) developed in Python [7] and BioPython [8]. MIA modules can be seen in Fig. 1, and it is capable of retrieving DNA sequences, and of calculating Entropy and Mutual Information spectra applying a statistical framework that allows inferences. This pipeline was developed due to the inexistence of an application able to calculate and display vertical Shannon entropy (VH), vertical mutual information (VMI), horizontal mutual information (HMI), and Jensen-Shannon Divergence (JSD) between pairs of different species spectra, herein called informational distances.

Entropy and Mutual Information theory can be found in [9, 10], and a nice review in [11]. Many applications [12–14] are capable of calculating biological sequence parameters, but only BioEdit [15] calculates mutual information and vertical entropy. There are also some theoretical studies like in Grosse et al. [1] focused on horizontal mutual information of DNA sequences, and other studies of co-evolution of proteins [3, 4, 16, 17] based on vertical mutual information. However none of these studies and applications has algorithms to calculate and display VH, VMI and HMI distributions and their informational distances.

Therefore, we began the development of MIA guided by the questions: Given some sequences, grouped in recognizable sets (species), are molecular data capable of discriminating theses sets using methods of information theory? Can we present statistical calculations that confirm or deny the results? Given a set of sequences with conserved and polymorphic residues and different lengths, how to deal with many possible alignments and their gaps?

In order to address these problems we demonstrated that Entropy and Mutual Information are good methods to deal with this complex problem, but caveats and warnings still remain: a) multiple sequence alignment (MSA) gives rise to many possible alignments herein denoted “mincut” for the minimum length alignment and “maxmer” for the maximum; b) gaps should be replaced by vertical consensus residues, differently from Weil et al. [4] who replaced them with a new character (a 21th amino acid) for their protein study, otherwise covariation between two residues will give rise to new states containing a strange fifth nucleotide; c) short strings present deviation called bias, thus we applied a bias correction for entropy, mutual information and respective standard errors as defined in Roulston [18] and also demonstrated by Steuer et al. in [11]; finally d) mutual information can be calculated residue by residue (1 x 1 positions) or 2 by 2 residues, or n by n residues - this parameter we will call NOL (number of letters of a word) and herein NOL will be equal to one (see Fig. 4b).

## Implementation

MIA has the following algorithms: A1) NCBI: gathers data at NCBI and stores them in gbk file format; A2) gbk to fasta: analyzes gbk file and organizes sequences in fasta files per species; A3) Alignment: aligns sequences with Muscle [19] and in the end creates two fasta files: “mincut” cutting out columns and sequences with large gaps and “maxmer” maintaining maximum number of gaps; A4) Purging: replaces ambiguous nucleotides via IUPAC nucleotide ambiguity table, and eliminates sequences with undesirable words in their names like “synthetic”; A5) Consensus: replaces gaps by their vertical consensus nucleotide; A6) VMI: calculates and stores Vertical Entropy (VH) and Vertical Mutual Information (VMI) spectra, and displays respective histograms and heat maps; A7) HMI: calculates and stores Horizontal Mutual Information (HMI) spectra, and displays histograms; A8) JSD: calculates Jensen-Shannon Divergence from pair of normalized spectra, storing distances and their SE in distance matrix files, and displays distance histograms; A9) HC: calculates hierarchical clusters and presents them as dendrograms, herein called informational dendrograms; A10) Entropy: simulates Shannon entropy.

Before gathering sequences it is important to analyze available Drosophila species data. There are some sites specialized in Drosophila data. They present sequence browsers, protein sequences, genes sequences and many parameters for molecular data. Three of these sites are: DPDB [20], Flybase [21] and BDGP [22].

In the first algorithm MIA is capable of searching for an organism, a gene or a word in the NCBI GBK. Here we searched for organism/genus “Drosophila” and the gene “Adh” (alcohol dehydrogenase). The resulting search retrieved 959 sequences of 291 species. From the total, 450 sequences of 17 species were selected (data gathered in March 2015). We did this task imposing an inferior limit called “number of sequence cutoff” in such a way that if this cutoff is high MIA finds a set with fewer species (there are not many genera/genes with a lot of sequences in NCBI). Otherwise, if the cutoff is low, the set will have a larger number of species, some of which with a low number of sequences. The consequence is that when calculating entropy and mutual information, a species with many sequences provides a lower standard error, while a species with fewer sequences provides a higher standard error. Therefore, in the Drosophila/Adh case we set the cutoff equal to 7 resulting in 17 species and 450 sequences.

After gathering sequences, the next step was the alignment algorithm - MIA uses Muscle for this task - and thereafter starts deleting columns and sequences “with many gaps” (which gives rise to the question – what is the “correct percentage of maximum gaps”?) replacing them by consensual vertical residues. However, deletions and replacements alter the informational distribution profiles. The human decision to set the percentage of possible gaps creates “mincut” and “maxmer” alignment sequences and their informational difference can be analyzed comparing distance matrices. Answering the question posed, “there is no correct choice” to how to deal with controlling gap deletions; only empirical tests and their results are likely ways to solve this problem in each genus/gene case.

With the aligned sequences MIA computes vertical entropy like did Adami in [9]. Thereafter MIA calculates mutual information in the horizontal direction as in Grosse et al. [1, 2, 23, 24], and in the vertical direction as in Martin et al. [16] and Hamacher et al. [3, 4]. All these methods are well explained in the Methods section that follows.

VH, HMI and VMI are calculated with and without bias correction; therefore the gain or loss of information for “mincut” versus “maxmer” with or without bias correction can be compared. Informational distances between different informational spectra are calculated via JSD method. Since JSD is not a linear function, standard errors are calculated by empirical propagation giving rise to distance matrices with SE.

ANOVA test was performed on each set of spectra for each method (VH, VMI, and HMI), in order to assess whether at least one spectrum was statistically different from the others (see Additional file 1: drosophila “summary” tab). Otherwise, all distributions would be statistically similar and we could not discriminate species. As can be noticed, this was only a first test to verify whether we could move forward.

All informational spectra were compared to spectra of shuffled and random sequences in order to analyze if they are statistically distinct. Spectra of shuffled sequence were generated using original sequences and shuffling the residues. Spectra of random sequence were created drawing nucleotides randomly, up to the same length as the original sequences. The first method preserves nucleotide contents and the latter is fully random tending to 25 % of representation to each nucleotide. In this study we will present only spectra of shuffled sequences and omit the random ones, since they presented similar results. Notwithstanding, MIA calculates and presents spectra for both methods.

### Methods

Shannon Entropy was defined in 1948 [25] as weighted average of the log of state probabilities,

$$ \mathrm{H}=-{\displaystyle \sum_{m=1}^k{\mathrm{p}}_{\mathrm{m}}\ast lo{g}_b\kern0.5em {\mathrm{p}}_{\mathrm{m}}} $$

Equation 1 – Shannon entropy.

In Equation 1 H is the Shannon Entropy, p_m_ is the probability of the existence of a state m in k possible states, and b is the base of the logarithm. If b is equal to 2 the entropic unit is defined as a “bit”, but if b is “e” (Neper’s number) the entropic unit is defined as “nat”, entropy derived from natural log. In this paper we will work only with “nat”.

Shannon Entropy of DNA sequences can be measured aligning sequences and calculating the relative frequency of encountering each nucleotide at determined residue (site). In this particular case only 4 possible states are found, Ω = {A, C, G, T}. Maximum entropy is defined as 1 MER [9], and it is achieved only when all states are equiprobable (p = 0.25). In this case *H*_*max*_^*DNA*^ = − ∑_*i* = 1_^4^ *p*_*i*_ log_2_ *p*_*i*_ = − 4 ∗ (1/4 ∗ log_2_1/4) = 2 bit or 1.386 nat. However, if one of the states has frequency equal to 1 (100 %), and the others are 0, the resulting entropy is H = 0 because log 1 = 0 and this residue is said to be conserved.

We shall call Vertical Entropy (VH) the way of calculating entropy counting nucleotide frequencies in the vertical orientation, that means, is a measure of how polymorphic any residue is after aligning sequences for determined species.

**Fig. 2:**
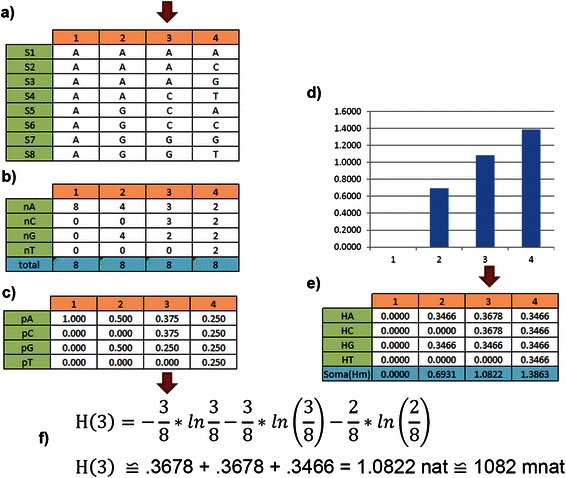
VH calculation: here we see an oversimplified DNA sequence alignment. The red arrow points to the residue (site) 3, but all residues are calculated: **a**) S1,S2 … to S8 are aligned sequences versus DNA positions (residues); **b**) nucleotide frequency distribution per residue; **c**) nucleotide relative frequency distribution per residue; **d**) vertical entropic spectrum; **e**) entropy per residue; and **f**) calculation of entropy for residue 3 or H[3]

In Fig. 2, we present an oversimplified alignment for 8 sequences: a) S1, S2 to S8 are 8 aligned sequences, i = {1,2,3,4} are nucleotide positions (or residues), each cell represents a nucleotide for a given sequence at a given position i; b) is the nucleotide frequency distribution per site for each residue; c) is the relative nucleotide frequency distribution per site; d) is the vertical entropic spectrum; e) are the values for each nucleotide entropic contribution and at the bottom is the vertical total entropy of each residue.

Thus, given the position i = 1, we realize that all nucleotides are “A”. For i = 2 we realize that 50 % of nucleotides are “A” and 50 % are “G”. As previously discussed, the first position has H = 0 nat and the residue is conserved, the second position has H = .693 nat and the residue is polymorphic. For i = 3, see the red arrow, we realize that this position is more polymorphic than the previous (see Fig. 2d) but less polymorphic than i = 4 that has H[4] = H_max_ = 1 MER.

Mutual Information (MI) represents the covariation between two random variables, here denoted X and Y [10, 11, 24]. Mutual Information (Equation 2) is defined by the sum of two entropies, in position i and j, minus the joint entropy H(i,j). As shown in the next two sections, MI can be applied in the Vertical direction of aligned sequences or in the Horizontal direction for one single or many sequences, aligned or not. Both calculations measure the nucleotide variability in two positions. The first position i is represented by the random variable X, and the second position j is represented by the random variable Y. Therefore, MI(X,Y) can be defined as,

$$ \mathrm{M}\mathrm{I}\left(\mathrm{i},\mathrm{j}\right)=\mathrm{H}\left(\mathrm{i}\right)+\mathrm{H}\left(\mathrm{j}\right)-\mathrm{H}\left(\mathrm{i},\mathrm{j}\right) $$

Equation 2 – Mutual Information between two positions (i, j).

Another parameter for MI calculation is the size of the word, whose width is defined as number of letters (NOL). Therefore, we can analyze co-variation between regions with width greater than 1. However, in this study we will calculate MI only for NOL = 1 (see Fig. 4b).

**Fig. 3:**
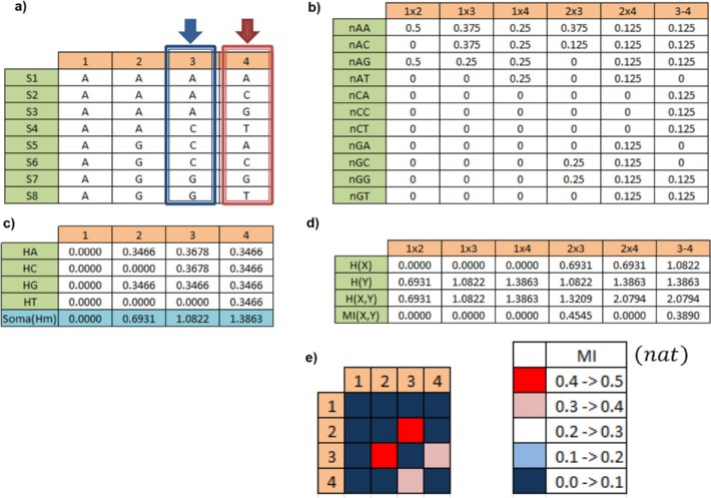
VMI calculation – the blue and red arrows point to a particular pair (X,Y), but all possible pairs are calculated: **a**) S1, S2 … to S8 are aligned sequences versus DNA positions (residues); **b**) nucleotide relative frequency distribution for all pairs of nucleotides; **c**) vertical entropy of each residue and at the bottom the vertical entropy per residue; **d**) mutual information calculation for pair of residues; and **e**) on the left is the VMI bidimensional spectrum represented as a heat map, and on the right side we see the color scale

In order to calculate the Vertical Mutual Information (VMI), see Fig. 3, we need first to estimate the nucleotide frequencies for position i and j, covering all residue pairs. Since, MI(X,Y) is a symmetric function, in other words, MI(X,Y) = MI(Y,X), and MI is zero if X covaries independently of Y, covering (i,j) in such a way that i = {1, L-1} and j = (i + 1, L), for j > i. VMI can also be expressed by,

$$ \mathrm{V}\mathrm{M}\mathrm{I}\left(\mathrm{i},\mathrm{j}\right)={\displaystyle \sum_{\mathrm{m},\mathrm{n}\kern0.5em \in \kern0.5em \left\{\mathrm{A},\mathrm{C},\mathrm{G},\mathrm{T}\right\}}\kern0.5em {\mathrm{p}}_{\mathrm{m}\mathrm{n}}\left(\mathrm{i},\mathrm{j}\right) \log \frac{{\mathrm{p}}_{\mathrm{m}\mathrm{n}}\left(\mathrm{i},\mathrm{j}\right)}{{\mathrm{p}}_{\mathrm{m}}\left(\mathrm{i}\right){\mathrm{p}}_{\mathrm{n}}\left(\mathrm{j}\right)}} $$

Equation 3 – Vertical Mutual Information.

In Equation 3 m and n are nucleotide states = {A,C,G,T}. Since we are talking about a bidimensional relationship, the resulting spectrum is represented by a heat map.

In Fig. 3, we present an oversimplified example of aligned sequence with the intention of explaining how to calculate VMI. The blue and red arrows point to a particular pair (X,Y), but all possible pairs are calculated where: a) S1, S2 … to S8 are 8 aligned sequences and i or j = {1,2,3,4} are nucleotide positions (or residues); b) is the nucleotide relative frequency distribution for all pairs of nucleotides; c) is the vertical entropy for each residue and at the bottom the vertical entropy per residue; d) is the mutual information calculation for pair of residues; and e) on the left is the VMI bidimensional spectrum represented as a heat map, on the right side is the color scale.

Horizontal Mutual Information (HMI) has a different concept and method of calculation when compared to VMI. HMI is defined as a measure of auto-covariation between two positions distant k units one from the other. Here k varies from 3 to L/2 (where L is the sequence length) with step equal to 1 in the 5′ to 3′ direction. The step is one, because we intend to calculate all residue to residue co-variations in the gene. In other words, transcription and translation rules are not necessary in our study.

For each value of k all sequence is covered counting all possible pair of nucleotides (m,n) Є{AA, AC, … TT}. Here, p_mn_ represents the probability to find a pair (m,n), where m and n Є {A,C,G,T}. HMI(k) is given by Equation 4, and p_m_ (k) and p_n_ (k) are marginal probabilities given by Equation 5 and Equation 6, respectively.

$$ \mathrm{H}\mathrm{M}\mathrm{I}\left(\mathrm{k}\right)={\displaystyle {\sum}_{\mathrm{m}=\left\{\mathrm{A},\mathrm{G},\mathrm{T},\mathrm{C}\right\}}{\displaystyle {\sum}_{\mathrm{n}=\left\{\mathrm{A},\mathrm{G},\mathrm{T},\mathrm{C}\right\}}{\mathrm{p}}_{\mathrm{m}\mathrm{n}}\left(\mathrm{k}\right)\ast \log \frac{{\mathrm{p}}_{\mathrm{m}\mathrm{n}}\left(\mathrm{k}\right)}{{\mathrm{p}}_{\mathrm{m}}\left(\mathrm{k}\right){\mathrm{p}}_{\mathrm{n}}\left(\mathrm{k}\right)}}} $$

Equation 4 – HMI equation for DNA sequences.

The marginal probabilities (p_m_(k)e p_n_(k)) can be calculated as,

$$ {\mathrm{p}}_{\mathrm{m}}\left(\mathrm{k}\right)={\displaystyle \sum_{\mathrm{n}=\left\{\mathrm{A},\mathrm{C},\mathrm{G},\mathrm{T}\right\}}{\mathrm{p}}_{\mathrm{m}\mathrm{n}}\left(\mathrm{k}\right)} $$

Equation 5 – Marginal probability of nucleotide m is p_m_ (*k*)

and

$$ {\mathrm{p}}_{\mathrm{n}}\left(\mathrm{k}\right)={\displaystyle \sum_{\mathrm{m}=\left\{\mathrm{A},\mathrm{C},\mathrm{G},\mathrm{T}\right\}}\kern0.5em {\mathrm{p}}_{\mathrm{m}\mathrm{n}}\left(\mathrm{k}\right)} $$

Equation 6 – Marginal probability of nucleotide n is p_n_ (*k*).

**Fig. 4:**
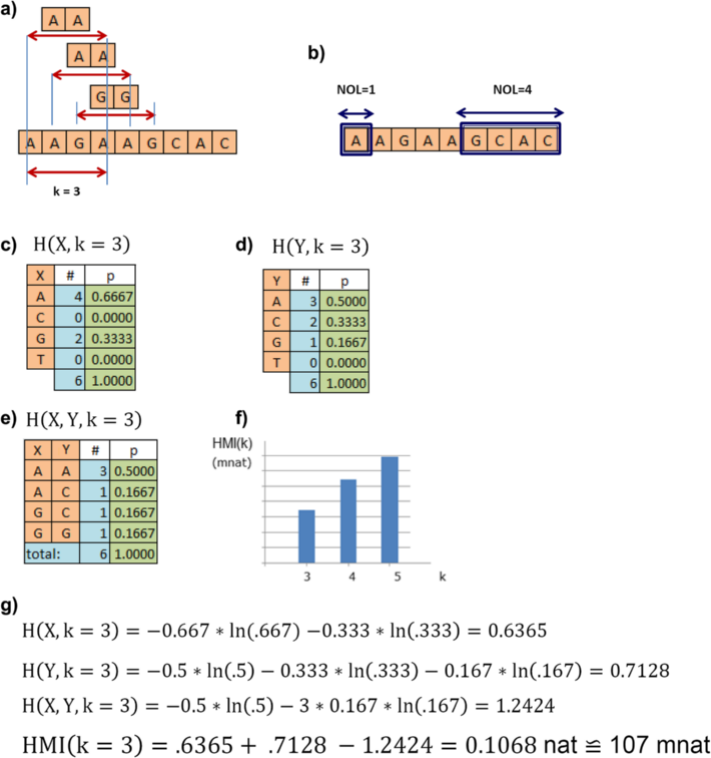
HMI calculation – **a**) k represents the distance between two residues ranging from 3 to L/2; **b**) is the number of letters, here NOL = 1; **c**) is the table of marginal frequencies of X for k = 3; **d**) is the table of marginal frequencies of Y for k = 3; **e**) is the table of frequencies for found pairs (X,Y); **f**) is the HMI spectrum; and **g**) is HMI calculation for k = 3

In Fig. 4, we present an oversimplified example of aligned sequence with the intention of explaining how to calculate HMI. In this example we see: a) k = 3 and the cursor covering the sequence from the left to the right finding = {AA, AA,GG…}; b) in this study NOL = 1, but NOL can be any other integer greater than 1; c) the marginal probabilities for X; d) the marginal probabilities for Y; e) the joint frequencies and relative joint frequencies for each found pair; f) HMI spectrum; and g) calculation of HMI for this particular case.

With all calculated spectra we used Jensen-Shannon Divergence (JSD) method to calculate the distances between all pairs of different informational species spectra, for VH, VMI and HMI. Since JSD needs two distributions to calculate a distance, we had to normalize the spectra and then apply them to this method. Therefore, we can calculate JSD to VH, VMI and HMI in order to calculate all distances between pairs of different species,

$$ \mathrm{J}\mathrm{S}\mathrm{D}\left(\mathrm{P}\left\Vert \mathrm{Q}\right.\right)=\mathrm{H}\left(\frac{\mathrm{P}+\mathrm{Q}}{2}\right)-\frac{1}{2}\left(\mathrm{H}\left(\mathrm{P}\right)+\mathrm{H}\left(\mathrm{Q}\right)\right) $$

Equation 7 – Jensen-Shannon Divergence (JSD).

Where P and Q are normalized spectra for different species. Equation 7 is the JSD equation, but the square root of JSD is indeed the distance between two distributions [26]. A distance equal to zero means that we cannot discriminate two distributions. A short distance means that both distributions are close and perhaps statistically impossible to discriminate depending on SE. Large distances means that species spectra are far allowing their discrimination, but also dependent on the SE. Thus, it can be inferred that JSD discriminates species with 95 % of probability if most of the distances do not fall in the confidence intervals (CI) of all the others – where CI ~ distance ± 2*SE.

## Results

We tested our algorithms searching in NCBI, at nucleotide database, for Organism = “Drosophila” and Gene = “Adh” (Alcohol Dehydrogenase) resulting in 959 sequences of 291 species. Only species with 7 or more sequences available were accepted to minimize the vertical entropy and mutual information standard errors. The final result was 450 sequences and 17 species, with lengths between 405 and 2204 bp. After going through the first two modules we encountered the Alignment module having 3 parameters which were designed to control column and line (sequences) gap deletions. The first parameter “Maximum Vertical Gaps1” (set to 10 %) allows gaps up to this percentage and transforms the data in minimum length sequences, or “mincut”. A maximum length sequence is obtained with the parameter “Maximum Vertical Gaps2” (set to 40 %) which allows more gaps, and whose resulting sequences are called “maxmer”. The third parameter “Maximum Horizontal Gaps” (set to 40 %) cuts out all sequences presenting more than 40 % of horizontal gaps. The resulting aligned sequences can be seen calling an external program called Seaview [12].

All aligned sequences resulted in “mincut” length equal to 588 bp and “maxmer” length equal to 859 bp. After this procedure, sequences were purged/filtered and not “ACGT” nucleotides replaced with their consensus via IUPAC [27] ambiguous table. Finally, consensus algorithm substitutes all gaps by the vertical consensus nucleotide.

VH, HMI and VMI were calculated for “mincut” and “maxmer” with and without bias correction. The final results presented twelve distance matrices, twelve distance histograms and twelve hierarchical cluster dendrograms (2 for mincut/maxmer × 2 for with/without bias correction × 3 informational methods). In order to perform these informational calculations we used NOL equal to 1.

According to the phylogeny proposed by van der Linde et al. [28] the subfamily *Drosophilinae* (subgenus *Sophophora* and subgenus *Drosophila*) shows that *D. paulistorum* is close to *D. willistoni* and *D. kikkawai* is fairly close to *D. melanogaster*. The first two are further away from the last two, and this was the choice to present our data in the following sections. Therefore, we will focus on these four species, only to summarize the explanations.

### Vertical entropy and mutual information

**Fig. 5:**
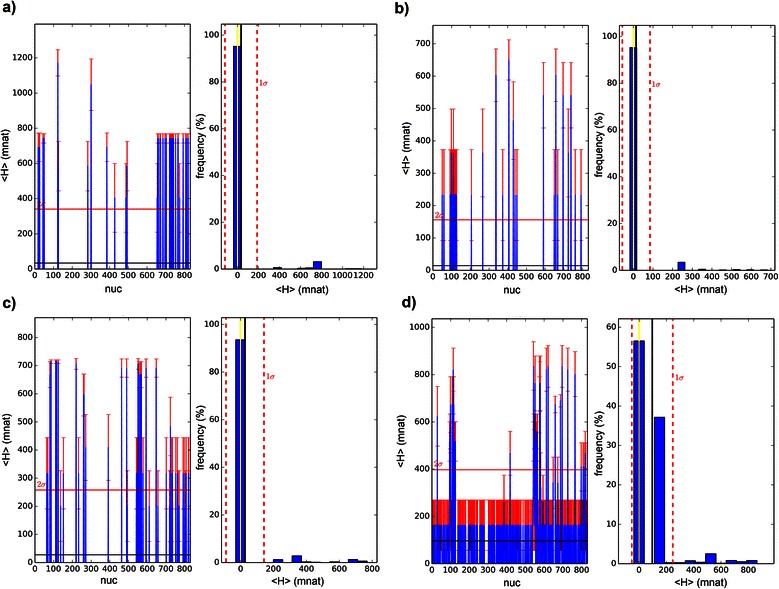
Vertical Entropy: four Vertical Entropy (in mnat) for maxmer sequences with bias correction, data from: **a**) *D. paulistorum*, **b**) *D. willistoni*; **c**) *D. kikkawai*; and **d**) *D. melanogaster*. On the left side, from each species frame, we see the entropic spectrum, vertical red lines are the SE(H_i_), where ‘i’ is the nucleotide position; the horizontal red line is 2 SD and the horizontal black line is the mean. On the right side we see the frequency distribution; in black is the mean, in red is 1 SD and in yellow is the median. Data are explained in text

VH and VMI were computed for each of the two positions (i,j) as in [4] and also explained in methods. VH spectrum can be seen in Fig. 5 and VMI spectrum – a heat map - in Fig. 7. Both, VH spectra and VMI heat maps can be visually discriminated. In order to assess whether at least one distribution is statistically different from the others, ANOVA test was performed and resulted in p-values near zero (for all maxmer/mincut versus with/without bias correction distributions). Therefore, there is at least one spectrum statistically different from all other spectra, and VH and VMI methods may be able to discriminate sets of molecular sequences.

Observing Vertical Entropy spectra (VH), in Fig. 5, the reader can visually discriminate the 4 frames with different profiles and frequency distributions. The data came from “maxmer” sequences with length equal to 859 bp, NOL = 1 and bias correction. Since the Adh locus is highly conserved (many residues with entropy equal to zero), the mean entropic value is very low and the standard error is large. On the left side of each frame we see the vertical red lines are SE(H(i)), where ‘i’ is the nucleotide position and SE(H) is the entropic standard error calculated from the polymorphism in this position over n sequences (species studies usually have different number of sequences). The horizontal red line stands for 2 standard deviations of the distribution and the black line for its mean. On the right side we see the frequency distribution graphic with 4 vertical lines: in black is the mean, in red is 1 standard deviation, in yellow is the median of the VH spectrum. Summarizing the four species: a) *D. paulistorum* has 12 sequences, mean(VH) = 37.7 (SD = 150.8) and median = 0 mnat; b) *D. willistoni* has 19 sequences, mean(VH) = 14.9 (SD = 70.8) and median = 0 mnat; c) *D. kikkawai* has 23 sequences, mean(VH) = 26.6 (SD =113.5) and median = 0 mnat; d) *D. melanogaster* has 30 sequences, mean(VH) = 96.7 (SD = 151.3) and median = 0 mnat.

**Fig. 6:**
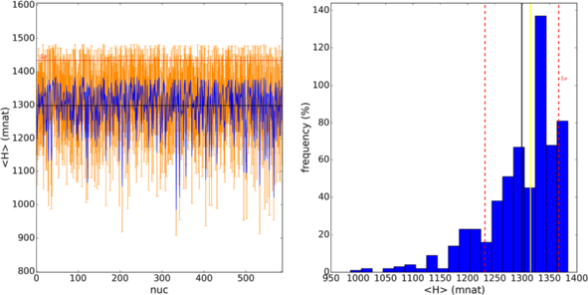
Vertical Entropy from shuffled sequences: “mincut” sequences (in mnat). On the left side we see the spectrum, in orange the SE and in blue the signal. On the right side we see the frequency distribution; in black is the mean, in yellow is the median and in red is 1 standard deviation. The “random” profile can be observed, very different from the *D. kikkawai*’s VH spectrum

Spectra of shuffled sequences simulated from original data can be seen in Fig. 6, having mean near 1 MER (maximum entropy), which produces a completely different spectrum when compared to the real data.

**Fig. 7:**
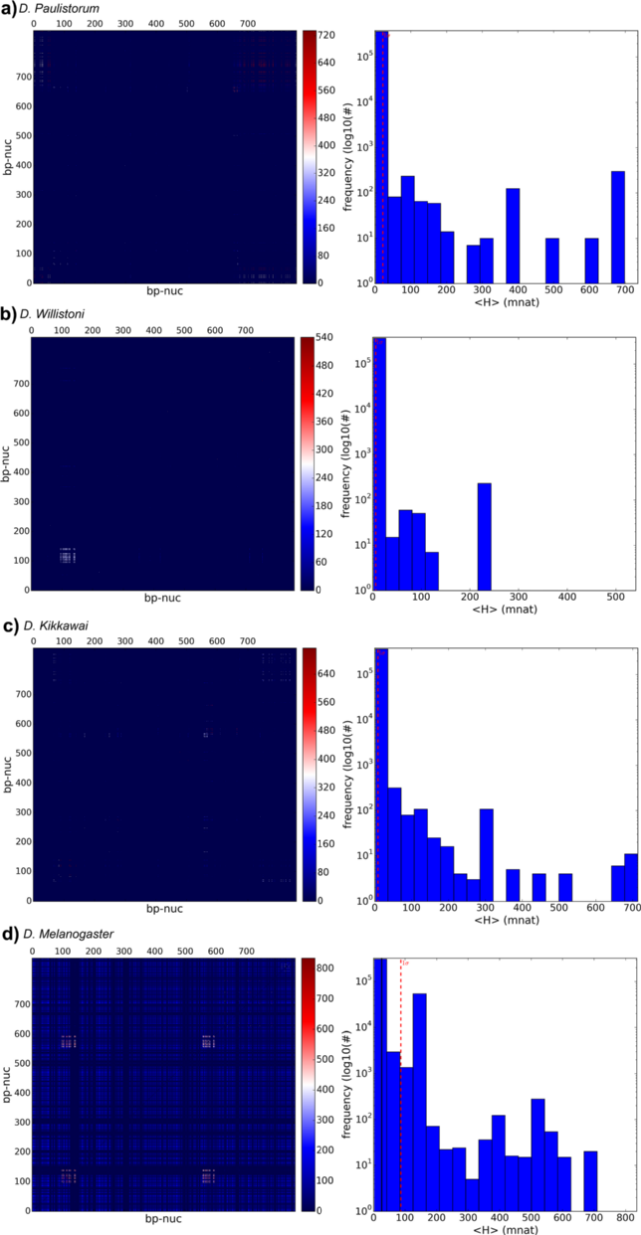
Vertical MI heat maps: four Vertical MI heat maps (in mnat) for maxmer sequences with bias correction from: **a**) *D. paulistorum*, **b**) *D. willistoni*, **c**) *D. kikkawai*, and **d**) *D. melanogaster*. Each heat map has a different profile and different maximum; data are explained in the text. The observation of peaks is possible by zooming the figure

Observing Vertical Mutual Information (VMI) spectrum, in Fig. 7, the reader can visually discriminate four heat maps with different patterns, different locations of peaks and different maximum values (zoom the image). All data came from “maxmer” sequences with length equal to 859 bp, NOL = 1 and bias correction: Summarizing the four species: a) *D. paulistorum* has 12 sequences, max(VMI) = 734.8 (SE = 104.6) mnat at a discreet point in the heat map (112 × 138 bp); b) D. willistoni has 19 sequences, max(VMI) = 541.0 (SE = 115.2) mnat at a discreet point in the heat map (712 × 754 bp); c) *D. kikkawai* has 23 sequences, max(VMI) = 713.9 (SE = 51.6) mnat close to a region with a bumpy profile localized at 89 × 117 bp; and d) D. melanogaster has 30 sequences, max(VMI) = 835.1 (SE = 120.5) mnat in a highly bumpy profile with maximum value at 637 × 712 bp.

**Fig. 8:**
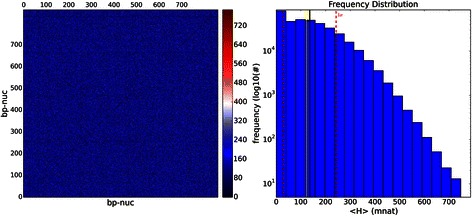
Vertical MI from shuffled sequences: VMI heat map in mnat for *D. kikkawai*. On the left side we see the heat map for shuffled each of the 23 sequence residues with maximum VMI = 841mnat. On the right side we see the frequency distribution, in black is the mean = 136 mnat, in yellow is the median = 122 mnat and in red is the standard deviation = 106 mnat. We concluded that all mutual information is lost. The “random” profile can be observed, very different from the *D. kikkawai*’s VMI

Spectra of shuffled sequences simulated from original data can be seen in Fig. 8, having mean = 136 mnat, a large SD = 106 mnat and median = 122 mnat, a completely different spectrum when compared to the real data. Here all mutual information is lost.

### Horizontal mutual information

HMI was computed as in [1] and also explained in methods. All 17 Horizontal MI spectra cannot be visually discriminated. In order to assess whether at least one distribution is statistically different from the others, ANOVA test was performed with all spectra and resulted in p-values near zero. Therefore, there is at least one spectrum statistically different from all other spectra, and HMI method may be able to discriminate sets of molecular sequences.

**Fig. 9:**
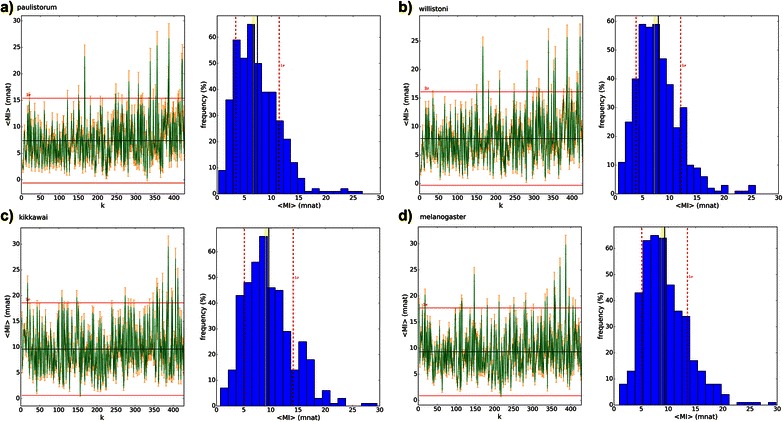
Horizontal MI spectrum: four Horizontal MI spectra (in mnat) for maxmer sequences and bias correction, data from: **a**) *D. paulistorum*, **b**) *D. willistoni*, **c**) *D. kikkawai*, and **d**) *D. melanogaster*. On the left side, from each species frame, we see the HMI spectrum, vertical orange lines are SE(HMI_k_), where k is the distance between positions; horizontal red lines are 2 SD and in horizontal black line is the mean. On the right side we see the frequency distribution; in black is the mean, in red is 1 SD, and in yellow is the median. Data are explained in text

Observing HMI spectra, presented in Fig. 9, the reader is unable to visually discriminate them, and that’s why JSD (see next section) is a method to measure informational distances between spectra. All data came from “maxmer” sequences with length equal to 859 bp, NOL = 1 and bias correction. On the left side of each of 4 species frames, we see HMI versus k distance, also called HMI spectrum. Each value HMI(k) has its own mean and standard error obtained from the ensemble (all sequences from determined set). The mean of HMI(k) (horizontal black line) and its standard deviation (the red line is 2 SD) can be observed. On the right side we see the frequency distribution for HMI spectrum with 4 vertical lines: in black is the mean, in red is 1 standard deviation, and in yellow is the median. Summarizing the four species: a) *D. paulistorum* has 12 sequences, mean(HMI) = 7.4 (SD = 4.1) and median = 6.6 mnat; b) *D. willistoni* has 19 sequences, mean(HMI) = 7.9 (SD = 4.1) and median = 7.2 mnat; c) *D. kikkawai* has 23 sequences, mean(HMI) = 9.6 (SD = 4.5) and median = 9.0 mnat; d) *D. melanogaster* has 30 sequences, mean(HMI) = 9.3 (SD = 4.2) and median = 8.8 mnat. All these mean values are very low and we certified that they are greater than the superior value from the confidence interval from shuffled analyses.

**Fig. 10:**
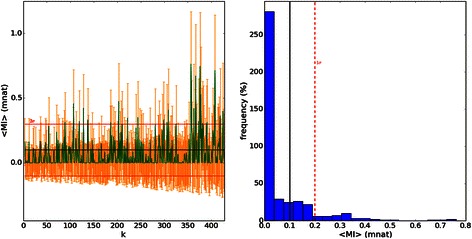
Horizontal MI spectrum from shuffled: “maxmer-bias correction” sequences (in mnat). On the left we see the spectrum, in orange the SE and in green the signal. The mean = 0.06 (0.12) mnat is pretty near zero, and median = 0.00 mnat. We conclude that all information is lost. The “random” profile near zero can be observed, very different from the *D. kikkawai*’s HMI spectrum. On the right side we see the frequency distribution; in black is the mean, in yellow is the median and in red is 1 standard deviation

Spectra of shuffled sequences simulated from original data can be seen in Fig. 10 having mean and median near zero mnat, a completely different spectrum compared to real data. Once again, all mutual information is lost. This is very important since all HMI have low mean, in a particular example, *D. paulistorum* has < HMI > = 7.2 mnat > > 0.063 (0.117) mnat for shuffled sequences. Therefore we can confirm that all spectra are statistically distinct compared to the spectrum of shuffled sequences. A nice discussion can be seen in [11, 29].

### JSD

**Fig. 11:**
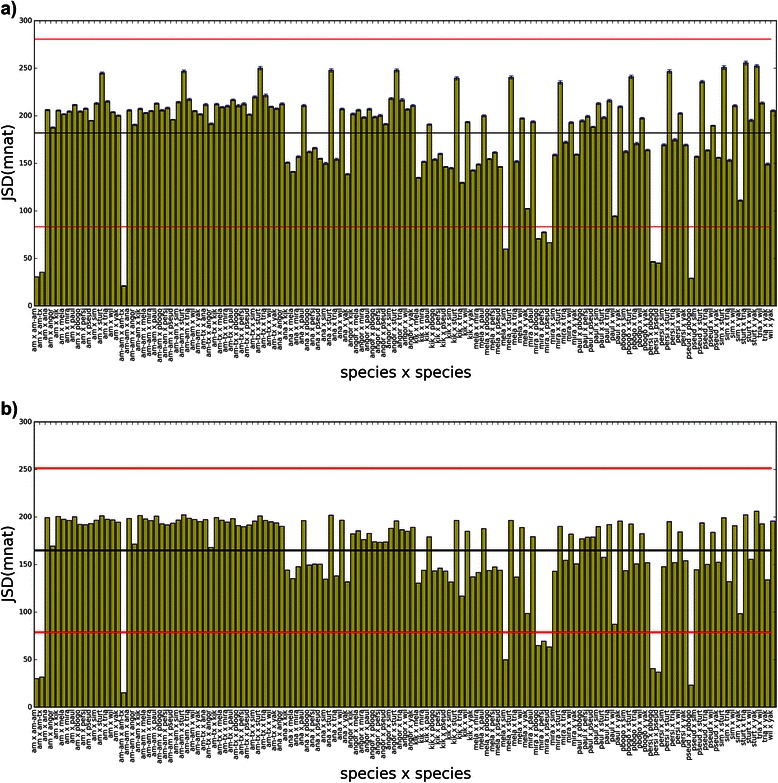
JSD histogram for HMI(maxmer): JSD histogram for pairs of HMI distributions for maxmer sequences **a**) with bias correction; and **b**) without bias correction: “am” for *D. americana*, “am-am” for *D. americana americana*, “am-tx” for *D. americana texana*, “ana” for *D. ananassae*, “angor” for *D. angor*, “kik” for *D. kikkawai*, “mela” for *D. melanogaster*, “mira” for *D. miranda*, “paul” for *D. paulistorum*, “pbogo” for *D. pseudoobscura bogotana*, “persi” for *D. persimilis*, “pseud” for *D. pseudoobscura*, “sim” for *D. simulans*, “sturt” for *D. sturtevanti*, “wil” for *D. willistoni*, “yak” for *D. yakuba.* The observation of species names and vertical error bars is possible by zooming the figure

**Fig. 12:**
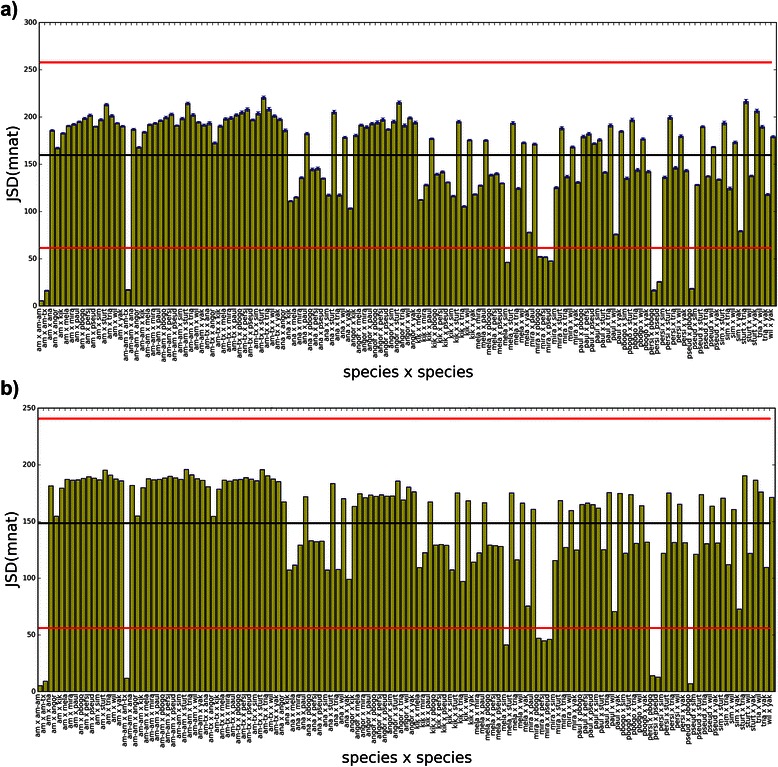
JSD histogram HMI(mincut): JSD histogram for pairs of HMI distributions for mincut sequences **a**) with bias correction; and **b**) without bias correction: “am” for *D. americana*, “am-am” for *D. americana americana*, “am-tx” for *D. americana texana*, “ana” for *D. ananassae*, “angor” for *D. angor*, “kik” for *D. kikkawai*, “mela” for *D. melanogaster*, “mira” for *D. miranda*, “paul” for *D. paulistorum*, “pbogo” for *D. pseudoobscura bogotana*, “persi” for *D. persimilis*, “pseud” for *D. pseudoobscura*, “sim” for *D. simulans*, “sturt” for *D. sturtevanti*, “wil” for *D. willistoni*, “yak” for *D. yakuba*. The observation of species names and vertical error bars is possible by zooming the figure

JSD applied to HMI spectra (JSD[HMI]) can be seen in Figs. 11 and 12, where informational distance histograms for maxmer and mincut, respectively, are displayed. Remarkable differences between distances are observed, many of them are statistically significant. Therefore, this method discriminates species with NOL = 1 and bias correction, since SE are negligible (see Additional file 1: HMI tab). However, phylogenetic studies are recommended to analyze whether closely related species are clusterized in a similar way.

**Fig. 13:**
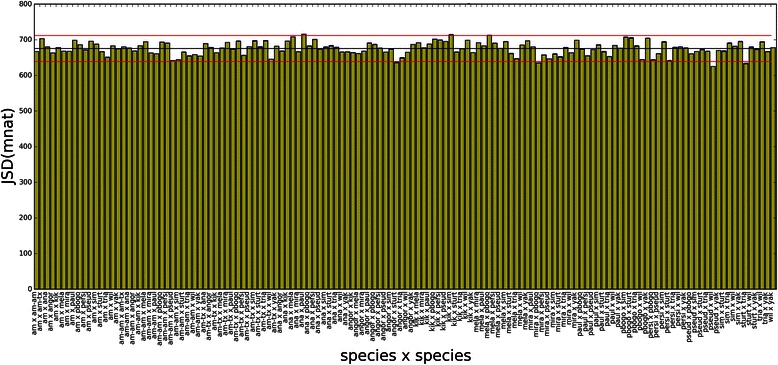
JSD histogram for HMI from shuffled sequences: maxmer with bias correction, mean = 675.6(18.2) mnat; “am” for *D. americana*, “am-am” for *D. americana americana*, “am-tx” for *D. americana texana*, “ana” for *D. ananassae*, “angor” for *D. angor*, “kik” for *D. kikkawai*, “mela” for *D. melanogaster*, “mira” for *D. miranda*, “paul” for *D. paulistorum*, “pbogo” for *D. pseudoobscura bogotana*, “persi” for *D. persimilis*, “pseud” for *D. pseudoobscura*, “sim” for *D. simulans*, “sturt” for *D. sturtevanti*, “wil” for *D. willistoni*, “yak” for *D. yakuba.* Very high distance with a very low SD can be observed for JSD[HMI] from shuffled sequences, demonstrating a regular profile very different from the original histogram

A histogram calculated from spectra of shuffled sequences can be seen in Fig. 13, having very high mean (680 mnat) and low standard deviation (17 mnat), a completely different profile when compared to the real histogram. This histogram shows the loss of the capacity to discriminate species, since the informational distances are very similar as well as the standard errors.

Observing VMI, most of informational distances have small differences. Furthermore, for VMI and VH respective standard errors are very large resulting in a large confidence intervals. These results imply that JSD applied to both methods results in not statistically significant distance differences. Therefore, VH and VMI poorly discriminate species with NOL = 1 and bias correction (see Additional file 1: VMI tab and VH tab).

### Hierarchical cluster

**Fig. 14:**
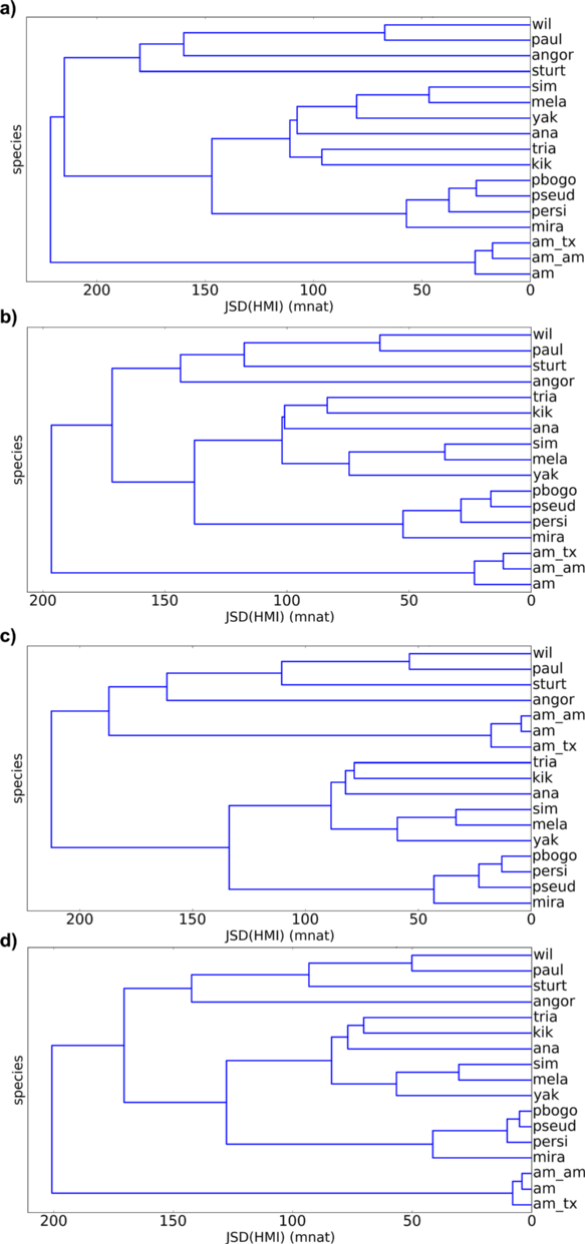
Hierarchical cluster dendrograms calculated for JSD[HMI], also called Informational Dendrogram: **a**) maxmer with bias correction; **b**) maxmer without bias correction; **c**) mincut with bias correction; **d**) mincut without bias correction. Acronyms are: “am” for *D. americana*, “am-am” for *D. americana americana*, “am-tx” for *D. americana texana*, “ana” for *D. ananassae*, “angor” for *D. angor*, “kik” for *D. kikkawai*, “mela” for *D. melanogaster*, “mira” for *D. miranda*, “paul” for *D. paulistorum*, “pbogo” for *D. pseudoobscura bogotana*, “persi” for *D. persimilis*, “pseud” for *D. pseudoobscura*, “sim” for *D. simulans*, “sturt” for *D. sturtevanti*, “wil” for *D. willistoni*, “yak” for *D. yakuba*

Hierarchical cluster analysis is the last algorithm and is computed based on a distance matrix calculated from JSD. In Fig. 14, we see four HMI dendrograms – here called Informational Dendrograms (ID) - obtained by applying weighted pair group with averaging method (WPGMA) for: a) maxmer with bias correction; b) maxmer without bias correction; c) mincut with bias correction; and d) mincut without bias correction. For JSD[VMI], applying bias correction, distances increase slightly and SE increases significantly (data not shown). But, for JSD[HMI], as seen in Figs. 11 and 12, standard errors are negligible and distances increase in few percentiles to 25 % when comparing mincut to maxmer. Those observed low SEs allow us to infer discrimination between species since distances are greater than zero with different values between pair of species. Changing from mincut to maxmer, with or without bias correction, some species change their positions in ID, but most of the clusters remain the same, like: “wil-paul-sturt-angor” (see acronym in the legend of Fig. 11), “tria-kik-ana”, “sim-mela-yak”, “pbogo-pseud-persi-mira”, and the “americanas” or “am-am_am-am_tx”. Because many of these species can be discriminated, these clusters must be compared to phylogenetic trees.

### Hierarchical cluster and phylogenetic gene trees

We also calculated phylogenetic trees for maxmer and mincut original sequences. We used Mega [14] for the methods Maximum Likelihood (ML) and Neighbor Joining (NJ). It was not our intention at all to compare phylogenetic trees versus mutual information dendrograms, but to observe whether ML and NJ can discriminate species for closely related species and whether the formed clusters are similar to the calculated informational dendrogram (ID) clusters.

**Fig. 15:**
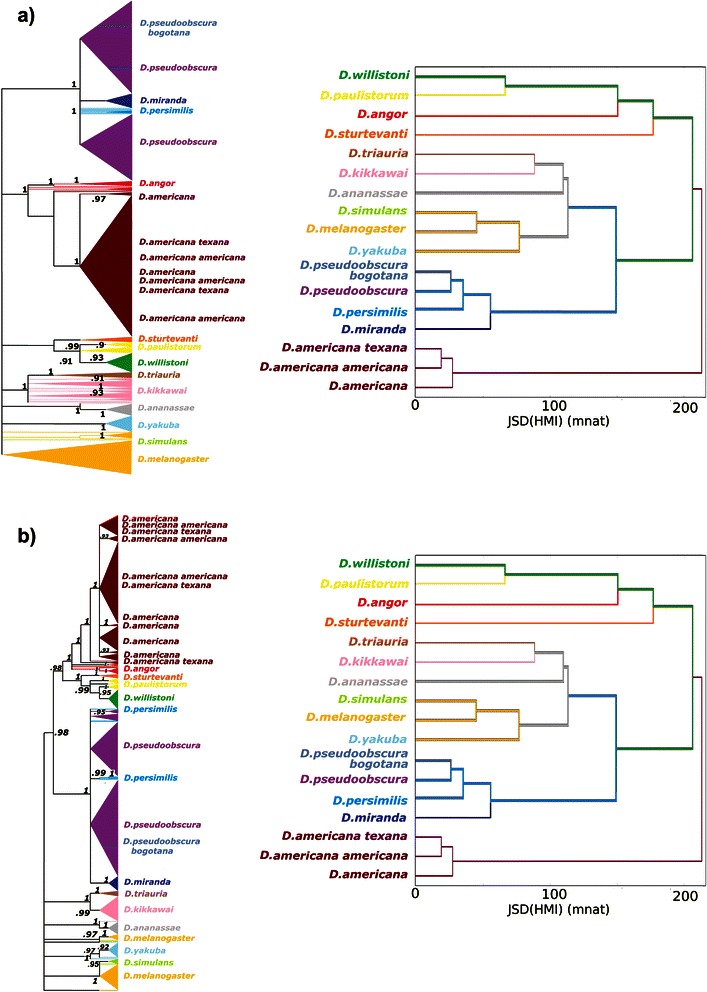
Phylogenetic Trees versus maxmer HMI-cluster: comparison between **a**) maximum likelihood versus informational dendrogram; **b**) neighbor joining versus informational dendrogram; ID was calculated from JSD[HMI | maxmer, bias correction]; bootstrap values can be observed at the nodes from ML and NJ

**Fig. 16:**
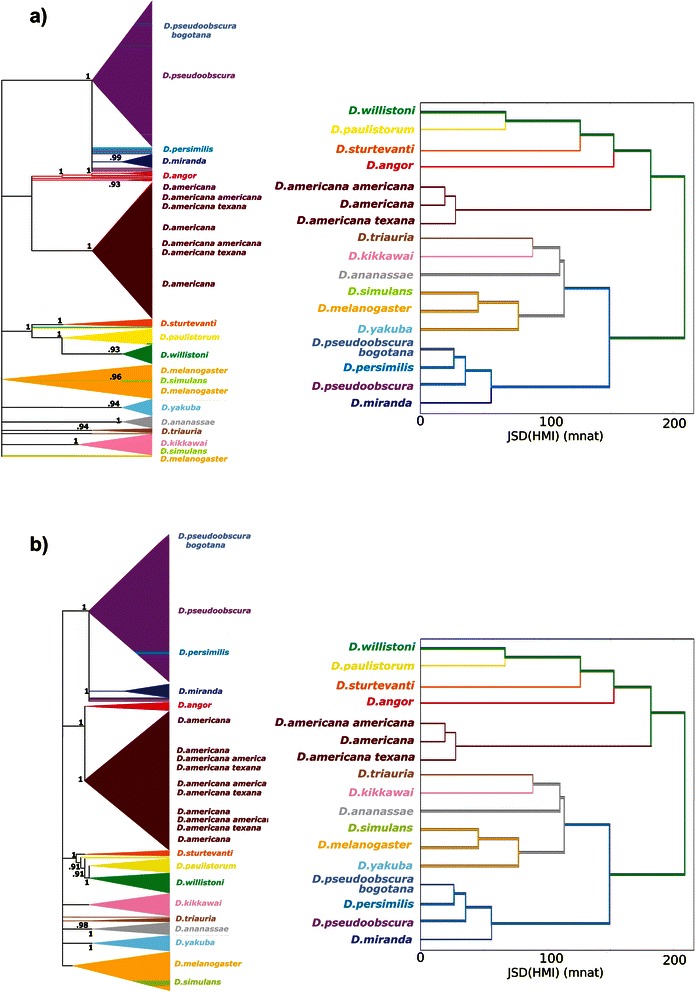
Phylogenetic Trees versus mincut HMI-cluster: comparison between **a**) maximum likelihood versus informational dendrogram; **b**) neighbor joining versus informational dendrogram; ID was calculated from JSD[HMI | mincut, bias correction]; bootstrap values can be observed at the nodes from ML and NJ

In Fig. 15, we see the gene tree for maxmer sequences - a) ML × ID, and b) NJ × ID. ML was calculated using TN93 model, the resulted max log likelihood (LnL) was −14561 and Ts/Tv equal to 1.30. In Fig. 16, we see the gene tree for mincut sequences - a) ML × ID, and b) NJ × ID. ML was calculated using TN93 model, the resulted LnL was −8004 and Ts/Tv equal to 1.53. One reason for different LnL is different lengths for maxmer and mincut sequences.

For maxmer sequences (Fig. 15), ML and NJ could not discriminate the “americanas” species (*D. americana*, *D. americana americana* and *D. americana texana*). The species in group “wil-paul-angor-sturt” (*D. willistoni*, *D. paulistorum,* and *D. sturtevanti*) are fairly close and this also occurs in JSD[HMI]-cluster method, except for *D. Angor*. This last group is close to the “americanas” cluster in NJ and ID. The species in group “sim-mela-yak” (*D. simulans*, *D. melanogaster* and *D. yakuba*) are close in all three methods. The species in group “tria-kik-ana” (*D. triaurium*, *D. kikkawai* and *D. ananassae*) are close in all three methods with a similar topology. And finally, the species in group “pbogo-pseud-persi-mira” (*D. pseudoobscura-bogotana*, *D. pseudoobscura, D. persimilis,* and *D. miranda*) are also close in all three methods.

## Discussion

Mutual Information refers to common variation between residues/sites, here DNA sequences. It can be applied to horizontal direction (HMI) and vertical direction (VMI). JSD can be applied to pairs of mutual information spectra representing “mutual informational distances”. These distances are used to infer discrimination between species. However, JSD applied to VH refers to “information distance”, like a difference of potential as pointed by Adami [9]. These three informational methods generated well defined spectrum patterns, similar to molecular signatures.

For HMI, mincut sequences resulted in almost the same distance profile when compared to maxmer (Figs. 11 and 12). Comparing Fig. 11: a) “with bias correction” and b) “without bias correction”, we observe that the profile gets bumpier, which is a nice feature that allows better discrimination between species. The same occurs in Fig. 12a, b.

VH and VMI spectra can be visually discriminated, as molecular signatures (Figs. 5 and 7), but it was more difficult to visually compare all HMI spectra (Fig. 9). However JSD, with respective SE, allowed us to infer that HMI can discriminate species (Figs. 11 and 12), while with VH and VMI methods it was not possible.

Shuffled tests applied to all information spectra and distance histograms confirmed the all original results are statistically distinct than the shuffled one.

Afterwards we compared Phylogenetic Gene Trees to Informational Dendrograms calculated for HMI spectra. In Figs. 15 and 16, we observed that the clusters and topologies are in reasonable concordance. But, we certified that these Informational Dendrograms (JSD[HMI]-clusterization) are not phylogenetic gene trees, they are only a mathematical way to cluster elements of the distance matrix.

## Conclusions

MIA is a user friendly pipeline capable in retrieving, selecting, aligning and storing molecular sequences. It is also capable in calculating Shannon Vertical Entropy, Vertical and Horizontal Mutual Information and JSD between these informational spectra. MIA exports fasta files, calculated spectrum files and distance matrices in ASCII format. It displays VH, HMI and VMI spectra. VMI heat maps can be visualized in 2D and 3D (not shown here). MIA also displays informational distance histograms and informational dendrograms. It is designed to analyze possible species discrimination via any molecular sequences, but in this first version only DNA sequences were analyzed. More tests must be done in a near future like increasing NOL for the same data and also more deep analyses like: polymorphic genes (not highly conserved), sequences with larger lengths, and many simultaneous gene analyses.

## Availability

MIA is freely available at https://github.com/flalix/MIA.

## Additional file

Additional file 1:
**Drosophila-Adh data.** (XLSX 153 kb)

## Abbreviations

JSD: Jensen-Shannon divergence
HC: Hierarchical cluster
HMI: Horizontal mutual information
HMI-cluster: Horizontal mutual information hierarchical cluster
Maxmer: Sequences with maximum gaps
MI: Mutual information
MIA: Mutual information analyzer
Mincut: Sequences with minimum gaps
MSA: Multiple sequence alignment
NOL: Number of letters or length of a word
VH: Vertical (Shannon) entropy
VMI: Vertical mutual information
